# Lessons learned from the experiences and perspectives of frontline healthcare workers on the COVID-19 response: a qualitative descriptive study

**DOI:** 10.1186/s12913-023-10062-0

**Published:** 2023-10-07

**Authors:** Marian Orhierhor, Wendy Pringle, Donna Halperin, Janet Parsons, Scott A. Halperin, Julie A. Bettinger

**Affiliations:** 1 Vaccine Evaluation Center, BC Children’s Hospital Research Institute, University of British Columbia, A5-950 West 28th Street, Vancouver, BC V5Z 4H4 Canada; 2 Department of Pediatrics, University of British Columbia, Vancouver, Canada; 3 Rankin School of Nursing, St. Francis Xavier University, 4130 University Ave, Antigonish, Nova Scotia B2G 2W5 Canada; 4 Canadian Center for Vaccinology, Dalhousie University, IWK Health, Nova Scotia Health, Halifax, Nova Scotia B3K 6R8 Canada; 5 Li Ka Shing Knowledge Institute, St. Michael’s Hospital, Unity Health Toronto, Toronto, Ontario M5B 1W8 Canada; 6 Department of Occupational Science & Occupational Therapy, Department of Physical Therapy, and the Rehabilitation Sciences Institute, University of Toronto, Toronto, Ontario Canada; 7 Departments of Pediatrics and Microbiology & Immunology, Dalhousie University, 5850/5980 University Ave, Halifax, Nova Scotia Canada

**Keywords:** Interviews, Healthcare workers, Healthcare settings, Health policy, Pandemic response, Pandemic preparedness, Future pandemic planning, BC response

## Abstract

**Background:**

During the COVID-19 pandemic, healthcare systems and healthcare workers (HCWs) faced significant demands and unique challenges. In this qualitative study, we explore the effects of the COVID-19 public health policies on British Columbia’s frontline HCWs, describe what worked in the management of the pandemic, and elucidate the lessons learned that could be applied to future pandemic preparedness, recovery and response.

**Methods:**

This qualitative descriptive study is part of a larger, national multi-case study on pandemic policy communication and uptake. Semi-structured interviews were conducted from November 2020- June 2021 with fourteen HCWs working in long-term care (LTC), acute care and public health settings. Data were inductively coded, and analyzed following a resilience framework for public health emergency preparedness, which emphasizes the essential elements of a public health system, vital to all phases of health emergency management, readiness, response and recovery.

**Results:**

HCWs experienced confusion, frustration, uncertainty, anxiety, fatigue and stress, during the pandemic and detailed challenges that affected policy implementation. This included communication and coordination inconsistencies between the province and regional health authorities; lack of involvement of frontline staff in pandemic planning; inadequate training and support; inadequate personal protective equipment resource capacity and mobilization; and staffing shortages. HCWs recommended increased collaboration between frontline staff and policy makers, investment in preparing and practicing pandemic plans, and the need for training in emergency management and infection prevention and control.

**Conclusions:**

Pandemic planning, response and recovery should include inputs from actors/key stakeholders at the provincial, regional and local levels, to facilitate better coordination, communication and outcomes. Also, given the critical roles of frontline HCWs in policy implementation, they should be adequately supported and consideration must be given to how they interpret and act on policies. Bi-directional communication channels should be incorporated between policymakers and frontline HCWs to verify the appropriate adoption of policies, reflective learning, and to ensure policy limitations are being communicated and acted upon by policy makers.

**Supplementary Information:**

The online version contains supplementary material available at 10.1186/s12913-023-10062-0.

## Background

Historically, previous public health emergencies and epidemics of HIV/AIDS, SARS, MERS, H1N1, Ebola and Zika have been catalysts for spurring discourse and actions needed for health care systems to improve emergency planning and response capacity [1–5]. As Khan et al., note, it is difficult to justify investment in emergency preparedness without an emergency to generate political and public attention [6]. For instance, outside of Asia, Canada was the region hardest hit by SARS in 2003, affecting the provinces of Ontario and British Columbia (BC), with the former reporting more morbidity and mortality [5, 7–9]. This prompted the need to strengthen the public health infrastructure that led to changes, such as the improvement of infection prevention and control in acute care hospitals, and the creation of the Public Health Agency of Canada, and Public Health Ontario, the latter of which was modeled after the BC Center for Disease Control [5, 7–9]. The recent COVID-19 pandemic provides another unique opportunity for jurisdictions to analyze their level of public health preparedness and response and implement changes to be better prepared for future events.

Health care workers (HCWs) are essential for responding to the effects of pandemics. Previous studies on the experiences of HCWs during the aforementioned outbreaks have shown that HCWs are at grave risk for contracting new and dangerous diseases, and they work under physical and psychological stress as they navigate new clinical and non-clinical challenges in the health care system [10–15]. These challenges include but are not limited to, increased workloads and staffing shortages, inadequate personal protective equipment (PPE), limited opportunities to engage with policymakers, lack of timely training on new procedures and protocols, inconsistent communication from multiple sources, lack of targeted and context-specific communication and frequent modification of infection control procedures and public health recommendations, which increase feelings of uncertainty among HCWs [10–15]. Many of the recommendations emanating from previous outbreaks have called for improved collaboration and communication between public health and primary care, increased workforce staffing and support, provision of training for redeployed staff and upscaling the knowledge and skills of HCWs in emergency management, infection prevention and control and pandemic preparedness plans [12, 15, 16].

Studies conducted in jurisdictions outside of BC during the first wave of the pandemic (December 2019-May 2020) suggest that HCWs directly treating COVID-19 patients and others working in hospital settings, still faced some of these challenges during the early response to the COVID-19 crises [17–21]. Given that these challenges seem prevalent, applying a context-sensitive approach to analyze public health capacities and responses as part of ongoing learning and preparedness for future crises is worthwhile to understand if experiences and challenges are similar or different across health settings [22]. This qualitative study explored the experiences and perspectives of BC frontline HCWs working in long term care (LTC), acute care and public health, to understand the impacts of and the responses to the pandemic public health policies during the first, second and third waves of the pandemic in BC. Our specific objectives were to 1) identify how HCWs responded to COVID-19 public health policies, 2) recognize what worked well in the management of the COVID-19 pandemic as well as challenges that arose, and 3) provide recommendations from the perspective of HCWs that can be useful for future pandemic planning and response.

## Methods

This study is part of a larger, national multi-case interpretive study investigating the impact of public health outbreak control policies on individuals and communities [23, 24]. For this study we focused specifically on the BC context, and used a qualitative descriptive approach to understand the experiences and perspectives of BC’s frontline HCWs during the COVID-19 pandemic [25–28]. Qualitative description lies within the naturalistic approach, allowing for a rich description of HCWs experiences using their own language and view points [25–28].

### Study setting, population and recruitment

The public health structure in BC comprises three administrative levels of organization: provincial, regional and local [29]. The provincial level is led by the provincial Public Health Officer (PHO), an independent position by law and the senior public health official in BC [30, 31]. The PHO is responsible for monitoring the health of the BC population and provides independent advice to the Ministry of Health and elected public officials on public health issues [29, 30]. At the regional level, there are five regional health authorities (HA) and a First Nations Health Authority, and each HA is led by a Chief Medical Health Officer who is responsible for delivering public health services within their respective regions [29]. HA also deliver services and programs ranging from primary health care, home care, to long term care and public health to various communities at the local level [29]. At the local level, a medical health officer oversees public health within a health service delivery region [29].

The health workforce in BC is comprised of about 25 designated health professions which are all subject to regulation by the Health Professions Act and 18 regulatory colleges [32]. In BC, more than 80% of the health workforce are women [33]. As at 2021, BC continued to struggle with long-standing health human resources shortages, especially in remote areas [34]. BC has about 26 physicians per 10,000 population (93.6% in urban areas; 6.2% in remote areas), and 77 nurses per 10,000 population, (94.3% in urban areas; 5.7% in remote areas) [34].

Under BC’s Public Health Act, the PHO is empowered to independently carry out certain executive functions, such as declaring a public health emergency and issuing orders that have the force of law [29–31]. The PHO sets the standards of practice and oversees the work of medical health officers [30]. The PHO led the response to the COVID-19 pandemic.

Recruitment was aimed at frontline HCWs working during the COVID-19 pandemic. We aimed to recruit at least twelve HCWs and defined frontline HCWs as those in leadership/administrative roles interpreting and implementing public health policies within their organization and HCWs in direct clinical roles or those who rendered other health services to members of the public within acute care, public health and LTC health settings. From November 2020- June 2021, fourteen English-speaking HCWs were recruited using a purposive sampling frame [25, 26, 35]. The sampling frame was established to recruit participants based on our definition of frontline HCWs as well as ideals of diversity among the target population representing a range of professional roles and healthcare settings. The research team identified participants through publicly available information (e.g., social media handle, institutional email, institutional phone number, or LinkedIn handle) and professional networks.

### Data collection

In adherence to physical and social distancing policies in place at the time of data collection, interviews were audio recorded and conducted over the phone or through video conferencing technology, according to participants’ preference; interviews lasted approximately 45-60 minutes. The interview guide (see additional file 1) was semi-structured and questions addressed the following topics: 1) participant understanding of COVID-19 public health policies at the provincial, regional, and local levels; 2) their experiences with and impacts of these policies at the individual level (in terms of how it affected their economic, health, social well-being); 3) perspectives on the broader impacts of these policies at the systemic level (institutions); 4) impact of policies on groups experiencing marginalizing conditions (for example individuals experiencing poverty/food insecurity, homelessness, and substance use); and 5) opinions on their agency and ability to influence or inform policy development and implementation. To ensure participants felt safe and respected in telling their stories during the interviews, we allowed participants to drive the conversation and answer questions/probes on the broad topics based on their personal experiences and what resonated with them, and in some cases participants spoke to some topic areas more than others.

There were three female interviewers for the study, one research staff and two graduate researchers, all trained in qualitative research. There was no relationship between interviewers and participants. To ensure data quality and unification of interview styles, the three interviewers participated in training on qualitative research and interviewing. This included a 60-minute training discussion on the practices of good qualitative interviewing with the principal investigators and readings on interview techniques and theories [36, 37]. During the research team biweekly meetings, interviewers had the opportunity to debrief on their interviews and share notes on narratives that emerged. This provided an avenue to identify recurring themes as well as emerging narratives that could be probed in subsequent interviews. Recruitment and interviewing were stopped when the research team felt there was information redundancy, meaning we were hearing nothing new after conducting 3 consecutive interviews. All interviews were transcribed verbatim, reviewed for accuracy and de-identified. Participants were provided with the opportunity to check their interviews for accuracy and privacy. The University of British Columbia Children's and Women's Health Centre of British Columbia Research Ethics Board (H20-02296) approved the study, and based on participants' preference, written or verbal informed consent was obtained before each interview.

### Data analysis

Data analysis followed a two-phase inductive-deductive sequence and began with familiarization of the complete set of transcripts by JAB, WP, and MO. Transcripts were then coded inductively with NVivo (version 13, QSR International) to identify passages relevant to the research questions, with two coders (MO and WP) independently generating initial codes for each of the first ten transcripts [38]. Codes and coded extracts were collated and compared by both coders. Similar codes were grouped together and conflicting codes were resolved after careful deliberations with each coder sharing their reflections, interpretations and criteria for assigning codes, and a best-fit code description was chosen with the consensus of MO, WP and JAB. A codebook with code names, code descriptions and examples was drafted, reviewed and revised during analysis meetings by JAB, WP and MO, and MO coded the remaining four transcripts with the finalized codebook.

Coded passages relating to our research questions about the response of HCWs to the COVID-19 public health policies, the successes and challenges of the pandemic response, and recommendations were then reviewed to identify key themes and patterns within the data. Theme definitions were drafted and revised by JAB, MO and WP during regular meetings to establish a best fit between data and research aims. (e.g., themes and definitions were reviewed and then split, regrouped, and revised as needed). During the process of defining themes, we observed commonalities with the Khan et al., resilience framework for public health emergency preparedness (PHEP) and proceeded to integrate this framework into our analysis. We compared and contrasted our emerging theme categories to seven relevant elements of the resilience framework to further inform the organization and interpretation of themes [6]. As such, themes were constructed inductively in the initial stages of familiarization and coding, and then deductively by way of the subsequent interpretation using the resilience framework [6, 38].

The framework examines eleven interacting essential elements of a resilient public health system that apply to all phases of health emergency management, readiness, response and recovery. We opted to utilize these elements to further inform our analysis, as they were developed in the Canadian context and are intended to assess practices and guide improvements in public health emergency preparedness and management. The essential elements include governance and leadership, planning process, collaborative networks, community engagement, risk analysis, surveillance and monitoring, practice and experience, resources, workforce capacity, communication and learning and evaluation. While it addresses eleven elements, we excluded four elements (risk analysis, community engagement, surveillance and monitoring, and learning and evaluation) in our analysis, as they were outside the scope of our research questions on the experiences of frontline HCWs.

## Results

**Fig. 1:**
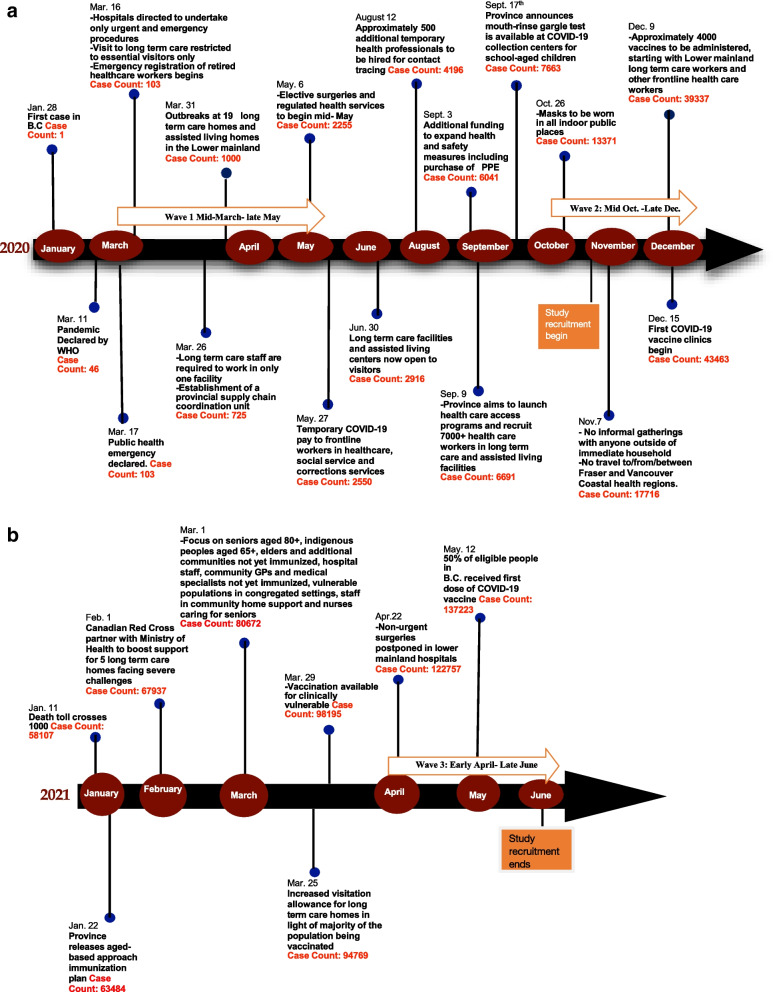
**a** A timeline of critical COVID-19 public health policies and events related to HCWs in British Columbia, Canada from Jan.-Dec. 2020. **b** A timeline of critical COVID-19 public health policies and events related to HCWs in British Columbia, Canada from Jan.-June 2021

Critical events and policies relating to HCWs in BC from January 2020 through June 2021 are shown in Fig. 1, with the recruitment time period also highlighted. A total of 14 HCWs were recruited and interviewed. The characteristics of HCWs are presented in Table 1. Participants included nurses, physicians, healthcare aides and social workers in LTC; public health nurses working in contact tracing, immunization, breastfeeding promotion and COVID-19 testing; and family physicians, nurses and other allied health staff working in acute care. As some participants described their organizational roles and responsibilities, there was fluidity in having leadership/administrative roles and providing direct healthcare or other clinical services to members of the public. We identified six major themes that describe the perspectives and experiences of HCWs.

**Table 1:** Demographic characteristics of participants

**Participant Characteristics**	***N* (%)**
**Health care setting**	
Acute care	4 (28.6)
Public health	4 (28.6)
Long term care/Home care	6 (42.8)
**HCWs type**	
Physicians	3 (21.4)
Nurses	8 (57.1)
Allied health professionals	3 (21.4)
(occupational therapist, social worker, healthcare aide)	
**Gender**	
Female	11 (78.6)
Male	3 (21.4)
**Employment status prior to March 2020**	
Full-time	10 (71.4)
Part-time	2 (14.3)
Casual	1 (7.1)
Retired	1 (7.1)
**Current employment status**	
Full time	13 (92.9)
Part-time	1 (7.1)
**Highest level of education**	
College/Diploma	1 (7.1)
Bachelors degree	8 (57.1)
Doctor of medicine	3 (21.4)
Masters degree	2 (14.3)
**Age (years)**	
25-34	6 (42.9)
35-44	1 (7.1)
45-54	3 (21.4)
55-64	3 (21.4)
65-74	1 (7.1)
**Years in Profession (years)**	
0-10	7 (50.0)
11-20	5 (35.7)
More than 20	2 (14.2)
**Years in current role (years)**	
0-2	8 (57.1)
3-5	3 (21.4)
More than 5	3 (21.4)

### Theme 1: governance and leadership in pandemic planning and response

Participants reflected on the importance of governance and leadership in facilitating an organized and effective pandemic response. They spoke on governance structures and the roles of the provincial government, the PHO and the HA in providing directives for the pandemic response. Two sub-themes emerged, i) the role of public health in pandemic planning and response and ii) Need for coordination among leadership levels.

i) **The role of public health in pandemic planning and response**

HCWs described clarity in the role, authority and responsibilities of the provincial public health leadership in the pandemic response. This was demonstrated by the perceived exemplary working relationship between public health leaders and politicians. They noted consistency in how the PHO, in particular, took the lead role in policy formulation and scientific decision making, and was the key spokesperson for public health policy while politicians supported this leadership and decision-making. Some acute care HCWs compared the public health leadership in BC favorably to that in other provinces, which they viewed to have strong political influences in scientific decision-making. They expressed gratitude for the public health leadership in BC and emphasized the importance of having the PHO as the single, respected, scientific voice and authority. Comparing the response during the 2003 SARS epidemic, a physician involved with both said:

*There were too many voices being the spokesperson for it [SARS 2003], and I remember they said, ‘What do you think that we could do differently?’ And I said, ‘What you could have done differently was have one reliable, scientific-based person doing the talking. Do not have a whole bunch of people, and especially do not have politicians because they’ve got no scientific basis. So I really think that—and I’m not the only one cause I mean, there’s lots of people that—you know, putting [the Provincial Public Health Officer] as the face and the voice of COVID here in B.C., um—was a huge thing. And I’ve had friends of mine from the States go, “wow, that’s just great”.* —*Physician*

ii) **Need for coordination among leadership levels**

In contrast to the exemplary comments around leadership by the PHO, participants criticized the lack of coordination between the message and policy being communicated at a provincial level and at the regional and local levels. Participants reported leadership among the various governance levels was uncoordinated at various time points throughout the pandemic, particularly around communication, adaptation and implementation of specific policies. HCWs gave examples of conflicting messages between the province and HA on masking, personal protective equipment and the availability of COVID-19 testing (Fig. 1a). The lack of alignment between provincial and regional policies created confusion at a local level about which guidelines to implement and how to do so. Long-term care HCWs gave examples of contrasting visitor policies at various facilities and noted messaging on visitation policies (Fig. 1a & b) was inconsistent across regions, which resulted in policies being interpreted differently at a local level in various LTC homes.

*There seemed to be a bit of barrier between what they were saying at the top and what was coming down to the care homes at the bottom, and which care homes are doing things differently, and [I] think that’s sometimes ‘cause their health authorities were giving them different rules to follow.* —*Allied health professional*

Some participants noted that in navigating these conflicting messages, their organizations had specific personnel who were tasked with interpreting policies to ensure their organizational procedures aligned with public health recommendations, and was feasible for their specific contexts, resources and workforce capabilities. HCWs advocated for increased co-ordination in leadership and communication between public health leaders at the provincial, regional and local levels, and noted this would allow for consistent messaging on public health interventions and recommendations.

### Theme 2: working together: planning processes and collaborative networks

Participants’ discussions about the pandemic planning process was closely linked to their comments about the underutilization of collaborative networks. They reported a lack of awareness at the regional and provincial level of the strengths and expertise of different groups and stakeholders within and outside the public health system that resulted in a lack of collaboration with frontline staff and underutilization of non-public health human resources which ultimately affected policy formulation and implementation.

Most HCWs critiqued the lack of collaboration with frontline staff and expressed dissatisfaction as their input was not being leveraged through the planning process at the provincial and HA level. It was unclear how and who made policy decisions directly affecting their job. Words like “higher-ups”, “top management”, “greater regional powers”, “top-down approach” were commonly used to describe the decision-makers and decision process and reflected their lack of engagement or consultation on the process and policies. Some HCWs emphasized the need for a more significant presence of their expertise and insights in policy formulation. Many participants believed their input could have led to a better understanding of the feasibility and impacts of policies and ensured better outcomes.

*They set up a new [COVID-19] testing site, for example, and instead of asking a couple of people that had worked at the other site a lot, as site lead, ‘Hey, we need to set up a new test site. What do you guys think about this location? What do you think about these tents? Like, this program, these staff?’. You know, there was no, ‘You’ve done this before. What do you think?’ They just, put tents up in a parking lot and then there were a bunch of problems. —Nurse*

The physician participants indicated they were sometimes consulted for their frontline expertise; however, they expressed frustration because their advice was sometimes ignored and when taken, their contributions were not acknowledged. In general, HCWs urged for timely collaboration with frontline staff and for their inputs to be acknowledged and implemented, or an explanation provided when their advice was not followed.

*Ask us for help early in the game, and take our suggestions seriously. ‘Cause we have a wealth of knowledge, that could help... I think inevitably, like, we’re all in this together. —Physician*

Participants further emphasized the need for public health to form early partnerships with other health care professionals to better leverage resources in the health care system and increase the efficiency of planning and response activities. A physician noted “it doesn’t all have to rest on public health’s shoulders. It can be done in coordination with family physicians, nurses, and many other healthcare professionals, especially, you know- IPAC [infection prevention and control] groups”. Physicians further gave examples of suspended child immunization programs and pointed out that public health should have been proactive in identifying health and social programs that needed extra staffing support due to redeployment of public health staff. They suggested that other non-public health care professionals with the required competencies could cover those vacant positions to ensure continuity. They applauded the approach of asking for volunteers to staff the mass COVID-19 vaccination clinics (Fig. 1a) and encouraged public health to always seek volunteers and expertise from other healthcare professionals in the future.

*Give a specific ask, you’ll get volunteers, gladly, but we can’t help if you don’t let us help.—Physician*

### Theme 3: practical learning and skill development

While the challenges of the pandemic provided rapid experiential learning, most HCWs felt more in-depth training was necessary and many noted they felt unprepared to take on new roles, manage emergencies and take on infection control practices in the early months of the pandemic.

Participants often questioned if their level of knowledge and skills needed for their roles as first responders in the pandemic were sufficient, and some felt the lack of adequate expertise reflected the lack of preparedness on the part of the provincial health system. They explained that many new plans and protocols had to be formulated and implemented “on the fly” within their organizations, and others thought the province lacked a pandemic preparedness plan.

*I think the biggest sort of learning here is that our system is not well designed for a pandemic. We just sort of had to put together a system on the fly… the Emergency Operations Committees, right, of the provincial sort of coordination. All, all of these kind of came about on the fly. We did not have plans for when pandemic hits.—Physician*

HCWs noted training support was late and not immediately available in the early months of the pandemic. They had to abruptly adopt new guidelines, take up additional roles or were redeployed to different departments, without adequate training. Referencing the lack of skills, experience and training needed for staff to work adequately in emergencies, a nursing coordinator gave an example below:

*None of the [regional or local] staff had been trained to use the [COVID-19 testing] program, so nobody knew how to work the program to, like, register people and print lab stickers and, all that stuff. And they didn’t have staff ready to work there, so they were redeploying acute care staff who worked at, the eye clinic for the last 20 years and have no idea how to do a nasopharyngeal swab, haven’t, worked in a fast-paced environment, just completely different background, super inconsistent, and new people everyday. So you’re training 10 new people how to do an NP [nasopharyngeal] swab, how to do the assessment, the flow of the site, the workflow, what’s required, all the infection control protocols, how to don and doff PPE [personal protective equipment]. People didn’t even know how to do that sometimes. And you’re doing that, every day at 8a.m, cars start coming through at 9am.*—*Nurse*

Although HCWs in LTC had previously experienced outbreaks of other infectious diseases such as influenza and Norwalk virus, robust infection control plans, training and practices were either lacking or not vigorous in these facilities. Some LTC workers were confused by the increasing layers of infection control guidelines and had to be trained or re-trained on infection control methods and how PPEs should be worn and used. HCWs called for increased investment in training in emergency management, and in training for new roles. LTC staff further advocated for the province and HA to set and implement higher standards of infection prevention and control practices in LTC sites, from the engineering of these sites to the education of staff.

*I felt that the healthcare workers [in LTC] really wanted this information [infection control] and in the past had been really left out of education. So healthcare workers can’t—you know, the healthcare aides cannot be left out —I think that the education of the staff in long-term care, from housekeeping all the way up to the registered nurses has to improve. There needs to be a higher standard of care that they have to adhere to* —*Nurse.*

Reflecting on their experiences from the first and second wave of the pandemic, HCWs across all settings saw the pandemic as a means of experiential learning for themselves and their organizations. They described experiencing an increased sense of community with colleagues, and growth in their professional competencies, feeling more confident in their knowledge, skills, and ability to adapt and efficiently perform their duties as the pandemic progressed. They also commended the public health leadership for their readiness to swiftly formulate and provide recommendations to reflect shifting realities as knowledge of the virus evolved. Some explained that the pandemic was an opportunity for the public health system to be better equipped for future pandemics and for pandemic plans to be written and tested, as the pandemic identified deficiencies and the areas needing improvement.

*So we’re all learning, so the pandemic playbooks are being written. We’ve learned so very much. - And we’re all set, ready for the next one. And unfortunately, we know there will be another one. So there—there’s no sense saying—thinking, well, this’ll never happen again. It’s going to happen again, but it will never happen like it did this time—we are better equipped now.*—*Allied health professional*

### Theme 4: availability and distribution of resources

HCWs felt the resources needed to adequately support their roles during the early pandemic response were insufficient. They critiqued the unavailability of secure supply chains for purchasing needed supplies, expressed displeasure at how resources such as PPE and the COVID-19 vaccines were distributed and noted that efforts to mobilize these resources could be improved.

There was a scarcity of PPE in the early months of the pandemic (Fig. 1a), with many organizations struggling to find and purchase it. Moreover, some health care organizations and provider types were prioritized. For example, acute care sites were prioritized for resource allocation of PPE and cleaning supplies. This created additional shortages in other organizations, such as LTC. A LTC nurse explained: “all the masks seemed to be going to the hospitals, all the cleaning supplies, all the resources seemed to be going to the hospitals too.” Due to the fear of further shortages, some LTC nurses reported that the management “hoarded” PPE and did not allow them to use their judgement to decide what patient care activities would require their use.

The uncertainties around the mode of COVID-19 transmission and combined PPE shortages created increased anxiety among HCWs, as they worried about having the proper protection to do their jobs safely. At some acute care sites, access to PPE became so tightly controlled that some allied health care professionals were restricted from using them.

*We did not have access to the hospital scrubs, and if we wanted some, they wouldn’t give them to us. I think, quite understandably, the rest of the OT [occupational therapy] team there blew their tops over this news because —what we were being told was that other professions can have access to PPE that we are not being given access to even though we’re seeing the same COVID-positive patients. — Allied health professional*

HCWs in leadership roles in acute care and LTC voiced their concerns about the lack of substantial information on secure supply chains from the HA and the province. This was a significant challenge, especially for older and rural institutions as they struggled to purchase PPE and other relevant supplies for the pandemic response.

*The emergency [departments] had to have these red and hot zones. But there wasn’t really ever clear direction about, like, who to go to for supplies. There could have been a lot more direction. Okay, we have a pandemic. Your hospital is old. It’s not set up to have isolation spaces as we’re planning or anticipating you need. Here’s a list of things that would be good for your site to order that should have been thought of decades ago*. —*Nurse.*

Participants viewed the COVID-19 vaccine as a vital resource for combatting the pandemic and generally had a positive perception of the vaccine rollout (Fig. 1a & b). Staff in LTC were grateful to be prioritized as this had a positive impact in reducing cases and outbreaks in their setting (Fig. 1a). Physicians applauded the mass vaccination clinics and virtual live translators, which ensured informed consent to community members with language barriers. Physicians further shared their thoughts on the vaccine roll out and suggested specific at-risk populations should have been prioritized for vaccination earlier. This included caregivers of the homebound elderly and certain healthcare provider groups (e.g. anaesthetists and surgeons) (Fig. 1b). Some stated improvements could have been made to the vaccine allocation to healthcare staff to speed the delivery of vaccines to appropriate populations. A LTC physician worried that special considerations for vaccinating the homebound elderly were not made. They felt it had not made sense to stop physicians from vaccinating their regular patients, when the physician would be the most trusted and familiar provider.

*Public health has said, the nurses at the community health centres will be the ones going out, doing the vaccinations [of homebound elderly], period. Nobody else. ... Why are we being so uh—like, even when the vaccine supply is good, I don’t understand why they’re being, only reliant on the nurses, and not, you know, the usual sort of—the docs who were doing the vaccinations. We [physicians] would do home visits. We would do the vaccine, right. Why not?*—*Physician*

### Theme 5: staffing shortages and enhancing existing workforce capacity

Participants commented on the uncertainty and longevity of the pandemic and the associated effects of staffing deficits and the impact this had on policy implementation and well-being of staff. HCWs who felt more supported by their organizations were better at managing the challenges posed by the pandemic than HCWs who felt less supported.

Many HCWs described feelings of frustration, fatigue, stress, burnout and increased pressure and anxiety due to additional duties, new roles, working long hours, and increased caseloads and shifts during the pandemic. It was noted that the health care system always lacked flexibility in staffing and this hindered the ability of staff to incorporate pandemic preparedness and response activities into their existing workloads.

*We constantly are working at full capacity. We create no slack in the system, both staff wise, system wise, and so anything that comes along, whether it’s a natural disaster or a pandemic. It throws everything into a loop because we just have no wiggle room.*—*Physician*

The lack of sufficient staff to support response activities was a challenge not only for public health contact tracing and childhood immunization teams, but also for rural hospitals and LTC sites that suffered from understaffing and underfunding prior to the COVID-19 pandemic. A nursing supervisor in a rural site stated, “We struggle on a busy day, never mind with a pandemic…and so when you talk about adding little bits of time to staff, we’re kind of already stretched to the max”. In LTC sites, the high ratio of residents to staff was aggravated by the pandemic, as staff had to work at only one site (Fig. 1a) to reduce the risk of COVID-19 transmission between sites. LTC staff reported reduced time and quality of care administered to each resident and difficulty managing residents with cognitive disabilities. They urged for increased staffing especially in LTC sites, so that workloads could be reduced for both nurses and care aides.

*Bringing a level of nursing hours and of care staff so that we don’t have to share, because we’ve learned through this pandemic, that sharing staff is detrimental to containing anything. Let’s get more staff so that way it—it helps our residents and it helps our workload. If there’s more staff, there’s less for me to do. [sighing] Now I can take a breath and actually spend some time with somebody.—Allied health professional*

Given the workforce shortages and other challenges that were experienced during the pandemic, sufficient organizational support was seen as an essential factor to help alleviate stress, build resilience and navigate challenges. Providing support to employees was seen not only as a source of motivation but it was also recognized as an important element for promoting a positive workplace culture to ensure HCWs wellbeing. Organizational support was characterized by participants in several ways including: providing training for new roles, financial incentives, provision of mental health and wellness support services, providing a clear outline of responsibilities and expectations, transparency and clear processes for redeployment. HCWs in acute care and public health further recommended that more structured processes could be employed when redeploying staff, such as considering seniority levels and establishing databases to track staff redeployment.

*I mean, if we were to do it again [redeployment to new roles], they’d probably be, like, a more structured rollout maybe. Like people getting signed off and, like, having, like, a better database in terms of, like, who is doing the signing off and who is getting signed off.—Nurse*

### Theme 6: perceptions on communication strategies

HCWs noted that the first few months of the pandemic were characterized by an overwhelming amount of information on policies and recommendations from the province and HA. They assessed the various communication platforms through which policies and recommendations were received and commented on the effectiveness of these channels in informing their response activities. Furthermore, they described how they reported implementation challenges, with many expressing dissatisfaction because formal feedback mechanisms were not available for voicing their concerns.

In discussing how they received information about policy changes and guidelines, HCWs described various information modalities. These included emails, internal team meetings, professional colleagues, town halls with HA and mass media sources, such as newspapers and the daily-televised press conferences of the PHO. Mass media and emails were the most common platforms for receiving information. Different HCWs' perceptions of the efficiency of email communication differed. LTC staff found emails to be a great way to communicate. However, HCWs in acute care and public health reported needing more time to read emails. A nurse leader in acute care shared, “nobody reads emails, I guess with frontline healthcare, you’re so busy for a 12 hour shift, and then when you do sit down you’re not necessarily opening your email.” They also found the frequency of emails overwhelming because policies were constantly updated or revamped, sometimes within hours. Participants agreed email communications were less organized at the beginning of the pandemic, but became more concise over time, making them more manageable.

*It was really confusing, ’cause you’d get a new email, like, two hours after you got to work and started…in the beginning… So now that I’m in public health, it’s still email, but it’s really clear and concise…I’d say now it’s way more organized than it was in March.—Nurse*

Participants commented on the mechanisms for providing feedback and voicing concerns when policies were communicated or implemented. Many HCWs commented they were unaware of opportunities to communicate feedback to provincial and HA-level policy makers. With the exception of HCWs who held medical leadership positions that interfaced with provincial and HA leaders, most participants felt that providing feedback was beyond their scope of influence and people above them mandated policies. Some acknowledged that a formal feedback process would be ideal, but with the pandemic, they felt this was not feasible due to time constraints and the rapidly evolving context. Others shared they usually could provide feedback informally to their managers or supervisors within their organizations but advocated for the process to be formalized. A nursing supervisor explained, *“*I think that the formal part might give the frontline staff a little bit more of an empowered voice. Like, that it’s not just something I told my supervisor and I don’t know what she did with it.” Physicians saw the feedback mechanism as an essential step. They noted that possible avenues for feedback were available for them, such as through the General Practice Services Commission, Doctors of BC and webinars conducted with the PHO. However, they also described that feedback mechanisms were very limited at the frontline and advocated for better avenues for two-way communication and discussion between frontline HCWs and policy makers

*There is no opportunity to have a discussion around them (policy decisions). It just seems like there’s no, access to...the people who sort of do the work day-to-day. The leadership sort of just kind of—they have their own, you know, place somewhere higher up that they just make their decisions and they’re just passed on and things just move on. At least if the centres, the regional centres, the local centres, the leads there, the infectious disease leads, the microbiology leads, right, they had a chance to sort of have a—you know, a two-way conversation with people who are making policy. I think that would be a great next step, because that’s where we’re stuck. Is we’re saying something, and we’re not getting any reception of it* —*Physician*

## Discussion

Our study of the experiences of HCWs in BC during the COVID-19 pandemic provides insights into how public health policies were understood and experienced by frontline HCWs in acute care, long term care and public health settings. HCWs reflected on and reported their experiences during the first, second, and third waves of the pandemic, and their accounts suggest changes that require attention at the provincial and regional levels to minimize challenges and improve policy implementation. Lessons learned from their experiences can inform policy and practice in preparation for future health crises in BC as well as other jurisdictions with comparable contexts.

Our account of HCWs experiences indicated key areas of weakness in the pandemic response. These included communication and coordination inconsistencies between the province and HA; lack of involvement of frontline staff in pandemic planning at the provincial and regional level; deficiencies in workforce capacity due to staffing shortages; lack of training and support; and inadequate resource capacity and mobilization. Some problems, such as the availability of PPE and the facilitation of training and support, were resolved during specific phases of the pandemic. Nevertheless, issues associated with coordination, collaboration, communication and staffing remained prevalent across the first, second, and third waves of the pandemic. Similarly, many of these issues arose in other parts of the world, as studies looking at the COVID-19 pandemic experience of HCWs revealed that HCWs struggled with inadequate PPE supplies, lack of appropriate training and support for redeployed staff, increased workloads, poor physical and mental wellbeing and frequent and sometimes contradictory changes in infection control procedures and public health recommendations [17, 19, 22]. Although these studies did not look at HCWs’ experiences specifically in BC, studies conducted in Ontario and Quebec have shown that HCWs in these Canadian provinces have also encountered similar difficulties [18, 20]. Unfortunately, some of the recommendations addressing these issues were noted in studies looking at lessons learned from SARS, H1N1, and MERS outbreaks, implying that these are persistent issues and there may be barriers to addressing them in some jurisdictions [12, 15, 16]. Given that these are recurring lessons and issues, it may be worthwhile for jurisdictions to plan for the future, and implement and evaluate recommendations to ascertain their fitness and relevance in improving pandemic preparedness and response.

### Investment in long-term care and public health

The 2003 SARS epidemic did not significantly affect LTC homes in Canada. Thus LTC sites have mostly been neglected in terms of pandemic planning, and many of the pre-existing challenges faced by LTC were exacerbated by the COVID-19 pandemic [9, 39, 40]. Many provinces across Canada were heavily affected during the pandemic with regard to LTC resident mortality, illness and staffing shortages [9, 39, 40]. This underscores the need to prioritize investments in LTC sites, with better infection prevention and control standards and deploying strategies, such as financial incentives, to train more health care aides, as observed in the province of Ontario, to address staffing shortages [41]. Also, the lack of investment in pandemic preparedness was reflected in Canada’s need to import PPE from other countries, which left HCWs in a vulnerable position and working in unsafe conditions [18, 42]. As demonstrated by many Asian countries during the pandemic, it is paramount to maintain and safeguard pandemic stockpiles of health supplies, and it is also necessary to provide organizations with directions on how to procure resources in the context of an emergency [43, 44]. This could include procurement protocols with guidance on identifying reliable suppliers, establishing and negotiating contracts, and other contingency plans when there are disruptions in the supply chain [43, 44]. Furthermore, for organizations to work innovatively with what is available in the context of limited resources, there needs to be established processes and transparency on allocating and using scarce resources during an emergency as this will likely ease tensions and reduce hoarding and mismanagement of supplies [45]. This might involve developing resource allocation protocols, in consultation with staff, that would outline how resources will be distributed, who makes the decisions and under what circumstances. Additionally, during emergencies, organizations could establish resource management teams with representatives from various departments to ensure no teams are unduly marginalized and to aid in comprehensive decision-making [45].

### Prioritizing collaboration and communication with stakeholders

Collaborations within and outside the public health system are fundamental for achieving successful outcomes. In this study, participants perceived that policy decisions were made at the top at the provincial and regional levels, without input from HCWs on the frontlines. This is similar to other studies that reported HCWs were not given avenues for feedback on pandemic policies [13, 20, 22]. Given the intensity of pandemics, we suggest that there is a need for bi-directional communication and transparency between policy makers and frontline HCWs, where important information such as the rationale, and the evidence behind policy decisions are shared with HCWs, to promote a climate of trust, mutual respect and support [13, 46]. Previous studies have also highlighted the significance of collaboration and communication between HCWs and policymakers [16]. However, they acknowledged that the lack of comprehension of the difficulties encountered by both parties during an epidemic or pandemic could be a barrier [16]. To overcome this gap, they propose that policy makers with experience working on the frontlines should be included in pandemic planning [16]. Healthcare workers (HCWs) in previous public health emergencies have observed they felt supported when there was a clear alignment and shared decision-making with policymakers [13]. Therefore, it could be beneficial to establish committees with representation from both HCWs and policy makers. When feasible, providing training sessions to policy makers and HCWs to enhance their comprehension of each other's challenges, roles, and perspectives could prove valuable.

Furthermore, although some participants described having feedback mechanisms, such as emails, team meetings and informal face-to-face conversations with supervisors within their organizations, they advocated for more opportunities and formalized processes. These process need to be in place prior to an emergency. Such formalized processes could include, dedicated town hall sessions with frontline staff for feedback, surveys, and web forms or other online platforms where feedback can be submitted. It might be crucial as well for formalized feedback mechanisms to cross jurisdictional levels, so regional and provincial policy makers are also learning from the frontlines. This may create opportunities for research and evaluation and facilitate the incorporation of lessons learned in ongoing responses. HCWs experiences also highlighted a need for optimum coordination and communication between collaborating partners and leaders at various levels. The uncoordinated leadership we identified affected how policies were communicated and implemented. Previous studies have highlighted partnership, coordination, and collaboration as vital components for ensuring efficient preparedness and response to public health emergencies and beyond, with the presence of coordination structures, infrastructure, roles, mandates, and adequate funding playing crucial roles as facilitators [47, 48]. Hence, we propose that coordination can be enhanced through clear reporting structures and well-defined communication protocols that outline responsibilities, expectations, and the manner in which information should be exchanged among partners at different levels. Additionally, fostering coordination could involve organizing regular forums and establishing centralized repositories where documents related to policies, guidelines, and best practices, can be made accessible to all stakeholders. Coordination and sharing of information across local, regional and provincial levels could prove to be a key measure to allow for policy limitations to be communicated, alignment of activities and messaging, as well as sharing expertise and insights [47, 48].

### Creating flexibility and increasing workforce capacity

The pandemic experience was portrayed as a means of experiential learning for many of our participants. In line with other studies, some HCWs felt a renewed sense of teamwork and support from their colleagues, and many felt more prepared, experienced and confident in their skills and knowledge as the pandemic progressed [13]. Those redeployed for new roles also felt supported and confident when they had sufficient skills and training for their new responsibilities. This further highlights the need for health care systems to be flexible, with adequate standard operating procedures and training manuals in place prior to an emergency, so new pandemic preparedness activities can be incorporated into the workloads of HCWs and these HCWs can be adequately supported in their new roles. HCWs generally perceived that there was a lack of preparedness; this might be because prior pandemic plans were inadequate due to the scope of the crisis or alternatively it may be a sign that operationalization and simulation of emergency plans in the frontlines were lacking [29]. Therefore, it could be vital to invest in testing and practicing relevant pandemic plans prior to any health crisis, in addition to incorporating continuous training modules on emergency management to enhance the competence of HCWs in terms of skills, knowledge and practical experiences.

### Strengths and limitations

Our study had several strengths. Interviews were conducted in the second to third wave of the COVID-19 pandemic in BC, allowing HCWs to examine what could have been done better early in the pandemic and consider what could be improved as the response moved forward. Secondly, in contrast with other studies focused on particular health care settings or HCWs type, this study highlights the perspectives and experiences of different types of HCWs in acute, public health and LTC settings. This provided the opportunity to probe for important emerging narratives to see if experiences and particular challenges were general or unique to specific health settings or HCWs types.

With respect to limitations, given the scope of our study and interview guide, four elements of Khan et al., PHEP framework could not be explored in this study, so other aspects vital to public health emergency preparedness and response were not reported [6]. It is important to note that recruitment occurred at a time when HCWs were heavily burdened with increased workloads thus resulting in a small sample size. As a result, HCWs in remote areas and other allied health professionals who may have different experiences were under-represented in this study. Thus, we would caution against generalizing the findings beyond the study population, however given the context, depths of perspectives represented and similarities with prior literature, lessons learned can be applied to similar jurisdictions. Future research could include quantitative surveys to explore how key themes uncovered in our study may be applicable to a larger number and more diverse group of HCWs.

Additionally, policy makers and decision makers at the provincial and regional level were not interviewed; thus we were only able to present the perspective of frontline HCWs. Many of the challenges and barriers they identified may not have, in fact, been overlooked at the higher leadership level. Even if these were just perceived challenges, it indicates better communication was needed with frontline staff. If there was an actual breakdown in co-ordination, we could not explore the upstream barriers that may exist in implementing proposed changes and the reasons for differential policy implementation at the local level. Thus, we propose these as important areas for future research with policy and decision-makers.

## Conclusion

The COVID-19 pandemic was a novel experience and a means of experiential learning for many frontline HCWs. The start of the pandemic was characterized by strict public health policies to control virus transmission. This was followed by frequent and regular policy changes as the pandemic rapidly evolved, causing uncertainty and confusion for HCWs. Reflecting on BC’s future pandemic preparedness, we suggest that stronger collaborations and partnerships be established between stakeholders within and outside the public health system and at the provincial, regional and local levels. Planning should include inputs from all levels, with feedback mechanisms incorporated to crosscheck the realities of policy implementation and ensure appropriate policy adoption and reflective learning.

### Supplementary Information

**Additional file 1. **Description of data: Semi-structured interview guide with topic areas and probes.

## Data Availability

The datasets generated and/or analysed during the current study are not publicly available due to containing information that could compromise research participant privacy and also due to the fact that permission was not sought at the time of participant interviews to share recordings or transcripts outside of the research team. For any enquiries regarding the datasets, kindly contact the corresponding author.

## Abbreviations

BC: British Columbia
HA: Health Authorities
HCWs: Health Care Workers
IPAC: Infection Prevention and Control
LTC: Long Term Care
PHO: Public Health Officer
PHEP: Public Health Emergency Preparedness
PPE: Personal Protective Equipment

## References

[1] Lurie N, Saville M, Hatchett R, Halton J (2020). Developing COVID-19 vaccines at pandemic speed. N Engl J Med..

[2] The Lancet Global Health (2022). The future of the International Health Regulations. Lancet Glob Heal.

[3] World Health Organization. Ebola then and now: Eight lessons from West Africa that were applied in the Democratic Republic of the Congo. https://www.who.int/news-room/feature-stories/detail/ebola-then-and-now (2019). Accessed 19 Oct 2022.

[4] Piret J, Boivin G (2021). Pandemics Throughout History. Front Microbiol..

[5] Health Canada. Learning from SARS: Renewal of public health in Canada-Report of the National Advisory Committee on SARS and Public Health; 2003. https://www.canada.ca/content/dam/phac-aspc/migration/phac-aspc/publicat/sars-sras/pdf/sars-e.pdf. Accessed 19 Oct 2022.

[6] Khan Y, O’Sullivan T, Brown A, Tracey S, Gibson J, Généreux M (2018). Public health emergency preparedness: A framework to promote resilience. BMC Public Health..

[7] Low DE. Learning from SARS: Preparing for the next disease outbreak: Workshop Summary. In: Knobler S, Mahmoud A, Lemon S, et al., editors. Institute of Medicine (US) Forum on Microbial Threats. Washington DC: National Academies Press (US); 2004. 22553895

[8] Webster P (2020). Canada and COVID-19: Learning from SARS. The Lancet..

[9] Silverman M, Clarke M, Stranges S (2020). Did lessons from SARS help Canada’s response to COVID-19?. Am J Public Health..

[10] Nanji KC, Orser BA (2015). Managing Ebola: Lessons Learned from the SARS Epidemic. Anesth Analg..

[11] Maunder R, Hunter J, Vincent L, Bennett J, Peladeau N, Leszcz M (2003). The immediate psychological and occupational impact of the 2003 SARS outbreak in a teaching hospital. Cmaj..

[12] Corley A, Hammond NE, Fraser JF (2010). The experiences of health care workers employed in an Australian intensive care unit during the H1N1 Influenza pandemic of 2009: A phenomenological study. Int J Nurs Stud..

[13] Billings J, Ching BCF, Gkofa V, Greene T, Bloomfield M (2021). Experiences of frontline healthcare workers and their views about support during COVID-19 and previous pandemics: a systematic review and qualitative meta-synthesis. BMC Health Serv Res..

[14] Chahley ER, Reel RM, Taylor S (2021). The lived experience of healthcare professionals working frontline during the 2003 SARS epidemic, 2009 H1N1 pandemic, 2012 MERS outbreak, and 2014 EVD epidemic: A qualitative systematic review. SSM - Qual Res Heal..

[15] Hodge J (2014). Canadian healthcare workers’ experiences during pandemic H1N1 influenza: Lessons from Canada’s response A review of the qualitative Literature.

[16] Desborough J, Dykgraaf SH, Phillips C, Wright M, Maddox R, Davis S (2021). Lessons for the global primary care response to COVID-19: A rapid review of evidence from past epidemics. Fam Pract..

[17] Aliyu S, Norful AA, Schroeder K, Odlum M, Glica B, Travers JL (2021). The powder keg: Lessons learned about clinical staff preparedness during the early phase of the COVID-19 pandemic. Am J Infect Control..

[18] Alami H, Lehoux P, Fleet R, Fortin JP, Liu J, Attieh R (2021). How can health systems better prepare for the next pandemic? Lessons learned from the management of COVID-19 in Quebec (Canada). Front public Heal..

[19] Ness MM, Saylor J, Di Fusco LA, Evans K (2021). Healthcare providers’ challenges during the coronavirus disease (COVID-19) pandemic: A qualitative approach. Nurs Heal Sci..

[20] Brophy JT, Keith MM, Hurley M, McArthur JE (2020). Sacrificed: Ontario healthcare workers in the time of COVID-19. New Solutions.

[21] Vindrola-Padros C, Andrews L, Dowrick A, Djellouli N, Fillmore H, Bautista Gonzalez E (2020). Perceptions and experiences of healthcare workers during the COVID-19 pandemic in the UK. BMJ Open..

[22] Chemali S, Mari-Sáez A, El Bcheraoui C, Weishaar H (2022). Health care workers’ experiences during the COVID-19 pandemic: A scoping review. Hum Resour Health..

[23] Yazan B (2015). Three approaches to case study methods in education: Yin Merriam and Stake. Qual Report.

[24] Stake RE (1995). The art of case study research.

[25] Bradshaw C, Atkinson S, Doody O. Employing a qualitative description approach in health care research. Glob Qual Nurs Res. 2017; 10.1177/2F2333393617742282. 10.1177/2333393617742282 PMC5703087 29204457

[26] Sandelowski M (2000). Focus on research methods: Whatever happened to qualitative description?. Res Nurs Health..

[27] Kim H, Sefcik JS, Bradway C, Nursing G (2017). Characteristics of qualitative descriptive studies: A systematic review. Res Nurs Health.

[28] Sullivan-Bolyai S, Bova C, Harper D (2005). Developing and refining interventions in persons with health disparities: The use of Qualitative Description. Nurs Outlook..

[29] Cheng M, Berman P (2022). Mapping the organization of British Columbia’s “Public Health System” and its adaptation to the COVID-19 crisis.

[30] Government of British Columbia. Office of the Provincial Health Officer. https://www2.gov.bc.ca/gov/content/health/about-bc-s-health-care-system/office-of-the-provincial-health-officer. Accessed 19 Oct 2022.

[31] Government of British Columbia. Public Health Act. https://www2.gov.bc.ca/gov/content/health/about-bc-s-health-care-system/legislation/public-health-act. Accessed 19 Oct 2022.

[32] BC Health Regulators. https://bchealthregulators.ca/health-regulation-in-bc/regulated-health-professions/. Accessed 11 Aug 2023.

[33] BC Women’s Health Foundation. Invisible No More: Inequities faced by women healthcare workers, especially during the COVID-19 pandemic, + recommendations for actions. 2021. https://assets.bcwomensfoundation.org/2021/11/29160837/BCWHF-invisiblenomore-report-full-FINAL-Nov29.pdf. Acessed 11 Aug 2023.

[34] Canadian Institute for Health Information. Health Workforce in Canada, 2021- Quick Stats. 2022. https://www.cihi.ca/en/topics/health-workforce. Accessed 11 Aug 2023.

[35] Patton MQ (1990). Qualitative evaluation and research methods.

[36] Kvale S (1996). The interview situation. Interviews: An Introduction to Qualitative Research Interviewing.

[37] Pezalla AE, Pettigrew J, Miller-Day M. Researching the researcher-as-instrument: An exercise in interviewer self-reflexivity. 2012. 10.1177/2F1487941111422107. 10.1177/1487941111422107 PMC4539962 26294895

[38] Miles MB, Huberman AM (1994). Qualitative data analysis: An expanded sourcebook.

[39] Liu M, Maxwell CJ, Armstrong P, Schwandt M, Moser A, McGregor MJ (2020). COVID-19 in long-term care homes in Ontario and British Columbia. CMAJ..

[40] Estabrooks CA, Straus SE, Flood CM, Keefe J, Armstrong P, Donner GJ, et al. Restoring trust: COVID-19 and the future of long-term care in Canada. FACETS. 2020; 10.1139/facets-2020-0056.

[41] Ministry of Long-Term Care. A better place to live, a better place to work: Ontario’s long-term care staffing plan (2021-2025). 2020. https://files.ontario.ca/mltc-ontario-long-term-care-staffing-plan-2021-2025-en-2020-12-17.pdf. Accessed 11 Aug 2023.

[42] Zhang J, Mitchell C, Kushniruk A, Guitouni A (2022). Facing disruption: Learning from the healthcare supply chain responses in British Columbia during the COVID-19 pandemic. Healthc Manag forum..

[43] Feinmann J. What happened to our national emergency stockpiles?. BMJ. 2021;10.1136/bmj.n2849. 10.1136/bmj.n2849 34848399

[44] Asian Development Bank. The Republic of Korea’s Coronavirus Disease Pandemic Response and Health System Preparedness. 2021. https://www.adb.org/publications/republic-korea-coronavirus-disease-pandemic-response. Accessed 11 Aug 2023.

[45] Lynch JF, Perera IM, Iwashyna TJ. Scarce resource allocation in a pandemic: A protocol to promote equity, timeliness, and transparency. Critical care explorations. 2021; 10.1097/2FCCE.0000000000000466. 10.1097/CCE.0000000000000466 PMC8189617 34124688

[46] Porat T, Nyrup R, Calvo RA, Paudyal P, Ford E (2020). Public health and risk communication during COVID-19-Enhancing psychological needs to promote sustainable behavior change. Front. Public Health..

[47] Gooding K, Bertone MP, Loffreda G, Witter S (2022). How can we strengthen partnership and coordination for health system emergency preparedness and response? Findings from a synthesis of experience across countries facing shocks. BMC Health Serv Res..

[48] Masotti P, Green ME, Birtwhistle R, Gemmill I, Moore K, O’connor K, et al. pH1N1-a comparative analysis of public health responses in Ontario to the influenza outbreak, public health and primary care: Lessons learned and policy suggestions. 2013; 10.1186/1471-2458-13-687. 10.1186/1471-2458-13-687 PMC3726397 23890226

